# Bis{2,4-dibromo-6-[(*E*)-(4-fluoro­benz­yl)imino­meth­yl]phenolato-κ^2^ *N*,*O*}zinc

**DOI:** 10.1107/S1600536812043930

**Published:** 2012-10-31

**Authors:** Dingjun Zhang, Hong Yu, Yue-Bao Jin, Ke-Wei Lei

**Affiliations:** a Lanzhou University of Technology, State Key Laboratory of Gansu Advanced Non-ferrous Metal Materials, Lanzhou 730000, Gansu Province, People’s Republic of China; b State Key Lab. Base of Novel Functional Materials and Preparation Science, Institute of Solid Materials Chemistry, Faculty of Materials Science and Chemical Engineering, Ningbo University, Ningbo 315211, People’s Republic of China

## Abstract

In the title Schiff base complex, [Zn(C_14_H_9_Br_2_FNO)_2_], the Zn^II^ atom is located on a twofold rotation axis and is coordinated by two O and two N atoms from two symmetry-related bidentate Schiff base ligands in a compressed tetrahedral geometry. The bond lengths and bond angles are within normal ranges. The dihedral angle between the least-squares planes of the aromatic rings within each ligand is 82.76 (17)°.

## Related literature

For the coordination ability of Schiff bases ligands, see: Rodriguez Barbarin *et al.* (1994 ▶). For photochromism and thermochromism in Schiff bases, see: Cohen (1964 ▶). 
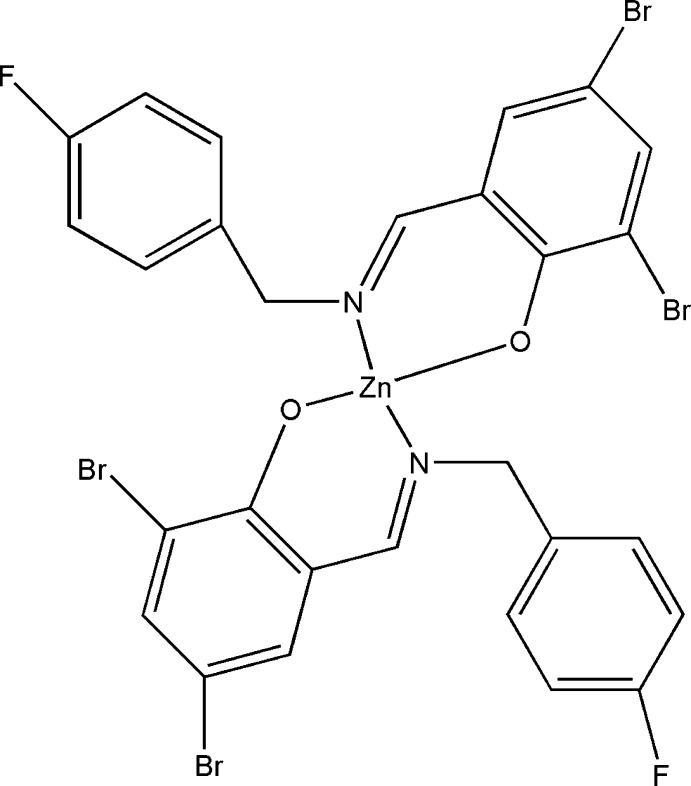


## Experimental

### 

#### Crystal data

[Zn(C_14_H_9_Br_2_FNO)_2_]
*M* *_r_* = 837.43
Monoclinic, 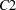

*a* = 14.6277 (11) Å
*b* = 9.8273 (3) Å
*c* = 13.0879 (10) Å
β = 133.490 (13)°
*V* = 1364.9 (3) Å^3^
*Z* = 2
Mo *K*α radiation
μ = 6.80 mm^−1^
*T* = 293 K
0.33 × 0.21 × 0.12 mm

#### Data collection

Rigaku R-AXIS RAPID diffractometer
Absorption correction: multi-scan (*ABSCOR*; Higashi, 1995 ▶) *T*_min_ = 0.197, *T*_max_ = 0.442
16865 measured reflections
2785 independent reflections
2733 reflections with *I* > 2σ(*I*)
*R*_int_ = 0.025

#### Refinement

*R*[*F* ^2^ > 2σ(*F* ^2^)] = 0.017
*wR*(*F* ^2^) = 0.041
*S* = 1.05
2785 reflections
177 parameters
1 restraint
H-atom parameters constrained
Δρ_max_ = 0.22 e Å^−3^
Δρ_min_ = −0.24 e Å^−3^
Absolute structure: Flack (1983 ▶), 1307 Friedel pairs
Flack parameter: −0.009 (7)

### 

Data collection: *RAPID-AUTO* (Rigaku, 1998 ▶); cell refinement: *RAPID-AUTO*; data reduction: *CrystalStructure* (Rigaku/MSC, 2004 ▶); program(s) used to solve structure: *SHELXS97* (Sheldrick, 2008 ▶); program(s) used to refine structure: *SHELXL97* (Sheldrick, 2008 ▶); molecular graphics: *SHELXTL* (Sheldrick, 2008 ▶); software used to prepare material for publication: *SHELXL97*.

## Supplementary Material

Crystal structure: contains datablock(s) global, I. DOI: 10.1107/S1600536812043930/ds2215sup1.cif

Structure factors: contains datablock(s) I. DOI: 10.1107/S1600536812043930/ds2215Isup2.hkl

Additional supplementary materials:  crystallographic information; 3D view; checkCIF report

## Figures and Tables

**Fig. 1.**
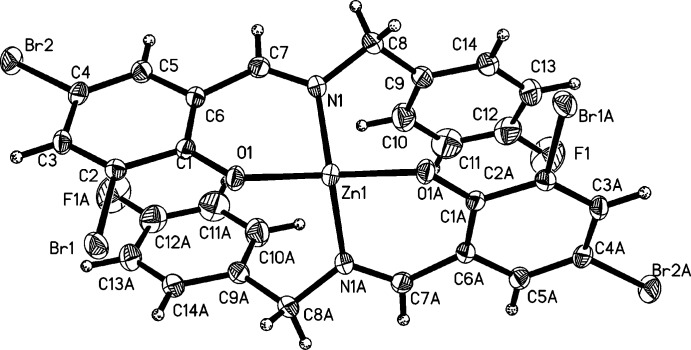
The structure of the title complex, showing 30% probability displacement ellipsoids and the atom-numbering scheme.

## supplementary crystallographic information

### Comment

Schiff bases ligands have been used with remarkable success in the field of coordination chemistry over past decades (Rodriguez Barbarin *et al.*, 1994). Schiff bases complexs show photochromism and thermochromism in the solid state by proton transfer from the hydroxyl O atom to the imine N atom (Cohen *et al.*,1964). Here we report on a new Schiff bases complex.

The molecular structure of title complex as illustrated in Fig.1. The dihedral angle between two benzenes rings is 83.281 (4)°. The Zn^2+^ atom is located on a twofold rotation axis in a compressed tetrahedral geometry and is is coordinated by two O atoms and two N atoms. The Zn1-O1 distance of 1.9311 (1)Å is shorter than the distance of Zn1-N1[2.0268 (4)Å](table 1) The bond lengths and bond angles in title complex are within normal ranges.

### Experimental

1 mmol(0.29g) of Zn(NO_3_)_2_ were added to 20 ml ethanol solution containing 2 mmol(0.77g) 2-((E)-(4-fluorobenzylimino) methyl)-4,6-dibromophenol.The resulting mixture was stirred for about 10 minute.The slow vaporisation of the solvent yielded after about 2 d yellow product.Yield:87.1%. Calcd.for C_28_ H_18_ Br_4_ F_2_ N_2_ O_2_ Zn: C,43.44;H,2.60;O,4.13;N,3.62; Found:C,43.50;H,2.61;O,14.15;N,3.61%

3,5-dibromo-2-hydroxybenzal dehyde(10mmol,2.80g) and (4-fluorophenyl)methanamine (10mmol,1.25g) dissolved in ethanol respectively. Then put them together and the solution was refluxed for 0.5h. After evaporation,a crude product was recrystallized twice from ethanol to give a pure yellow product.

### Refinement

All H atoms were placed in geometrically idealized positions and constrained to ride on their parent atoms (C—H = 0.93 %A) and Uiso(H) values equal to 1.2 Ueq(C).

### Crystal data

**Table:**

[Zn(C_14_H_9_Br_2_FNO)_2_]	*Z* = 2
*M**_r_* = 837.43	*F*(000) = 808
Monoclinic, *C*2	*D*_x_ = 2.038 Mg m^−^^3^
Hall symbol: C 2Y	Mo *K*α radiation, λ = 0.71073 Å
*a* = 14.6277 (11) Å	θ = 2.8–26.4°
*b* = 9.8273 (3) Å	µ = 6.80 mm^−^^1^
*c* = 13.0879 (10) Å	*T* = 293 K
β = 133.490 (13)°	Block, yellow
*V* = 1364.9 (3) Å^3^	0.33 × 0.21 × 0.12 mm

### Data collection

**Table:**

Rigaku R-AXIS RAPID diffractometer	2785 independent reflections
Radiation source: fine-focus sealed tube	2733 reflections with *I* > 2σ(*I*)
Graphite monochromator	*R*_int_ = 0.025
ω scans	θ_max_ = 26.4°, θ_min_ = 2.8°
Absorption correction: multi-scan (*ABSCOR*; Higashi, 1995)	*h* = −18→18
*T*_min_ = 0.197, *T*_max_ = 0.442	*k* = −12→12
16865 measured reflections	*l* = −16→16

### Refinement

**Table:**

Refinement on *F*^2^	Secondary atom site location: difference Fourier map
Least-squares matrix: full	Hydrogen site location: inferred from neighbouring sites
*R*[*F*^2^ > 2σ(*F*^2^)] = 0.017	H-atom parameters constrained
*wR*(*F*^2^) = 0.041	*w* = 1/[σ^2^(*F*_o_^2^) + (0.0207*P*)^2^ + 0.6898*P*] where *P* = (*F*_o_^2^ + 2*F*_c_^2^)/3
*S* = 1.05	(Δ/σ)_max_ = 0.002
2785 reflections	Δρ_max_ = 0.22 e Å^−^^3^
177 parameters	Δρ_min_ = −0.24 e Å^−^^3^
1 restraint	Absolute structure: Flack (1983), 1307 Friedel pairs
Primary atom site location: structure-invariant direct methods	Flack parameter: −0.009 (7)

### Special details

**Table:**

Geometry. All esds (except the esd in the dihedral angle between two l.s. planes) are estimated using the full covariance matrix. The cell esds are taken into account individually in the estimation of esds in distances, angles and torsion angles; correlations between esds in cell parameters are only used when they are defined by crystal symmetry. An approximate (isotropic) treatment of cell esds is used for estimating esds involving l.s. planes.
Refinement. Refinement of F^2^ against ALL reflections. The weighted R-factor wR and goodness of fit S are based on F^2^, conventional R-factors R are based on F, with F set to zero for negative F^2^. The threshold expression of F^2^ > 2sigma(F^2^) is used only for calculating R-factors(gt) etc. and is not relevant to the choice of reflections for refinement. R-factors based on F^2^ are statistically about twice as large as those based on F, and R- factors based on ALL data will be even larger.

### Fractional atomic coordinates and isotropic or equivalent isotropic displacement parameters (Å^2^)

**Table:**

	*x*	*y*	*z*	*U*_iso_*/*U*_eq_	
Br1	1.04547 (2)	0.46777 (3)	1.37836 (3)	0.04434 (8)	
Br2	0.66508 (3)	0.09397 (3)	1.20503 (3)	0.05004 (8)	
Zn1	1.0000	0.30224 (4)	1.0000	0.03419 (10)	
F1	1.0457 (3)	−0.1547 (3)	0.6672 (3)	0.1080 (9)	
O1	0.97414 (16)	0.36983 (17)	1.11706 (18)	0.0347 (4)	
N1	0.83087 (19)	0.2066 (2)	0.8654 (2)	0.0366 (5)	
C1	0.9037 (2)	0.3095 (2)	1.1302 (2)	0.0281 (4)	
C2	0.9183 (2)	0.3416 (2)	1.2459 (3)	0.0310 (5)	
C3	0.8483 (2)	0.2810 (3)	1.2690 (3)	0.0346 (5)	
H3	0.8609	0.3047	1.3467	0.042*	
C4	0.7589 (2)	0.1838 (3)	1.1733 (3)	0.0360 (5)	
C5	0.7374 (2)	0.1507 (3)	1.0573 (3)	0.0356 (5)	
H5	0.6754	0.0874	0.9933	0.043*	
C6	0.8086 (2)	0.2117 (2)	1.0336 (3)	0.0314 (5)	
C7	0.7726 (2)	0.1769 (3)	0.9027 (3)	0.0364 (5)	
H7	0.6986	0.1271	0.8384	0.044*	
C8	0.7676 (2)	0.1760 (3)	0.7192 (3)	0.0445 (6)	
H8A	0.7485	0.2608	0.6700	0.053*	
H8B	0.6883	0.1305	0.6725	0.053*	
C9	0.8450 (2)	0.0878 (3)	0.7088 (3)	0.0373 (5)	
C10	0.9129 (3)	−0.0210 (4)	0.7977 (3)	0.0583 (8)	
H10	0.9133	−0.0392	0.8677	0.070*	
C11	0.9809 (4)	−0.1039 (4)	0.7839 (5)	0.0726 (11)	
H11	1.0268	−0.1776	0.8438	0.087*	
C12	0.9785 (3)	−0.0746 (4)	0.6806 (4)	0.0613 (9)	
C13	0.9123 (3)	0.0310 (4)	0.5906 (3)	0.0547 (8)	
H13	0.9120	0.0479	0.5205	0.066*	
C14	0.8450 (2)	0.1132 (3)	0.6051 (3)	0.0419 (6)	
H14	0.7993	0.1863	0.5443	0.050*	

### Atomic displacement parameters (Å^2^)

**Table:**

	*U*^11^	*U*^22^	*U*^33^	*U*^12^	*U*^13^	*U*^23^
Br1	0.04587 (15)	0.05435 (16)	0.03650 (13)	−0.01934 (12)	0.02976 (12)	−0.01577 (12)
Br2	0.05293 (16)	0.06223 (18)	0.05420 (17)	−0.01660 (14)	0.04423 (15)	−0.00359 (13)
Zn1	0.0318 (2)	0.0480 (2)	0.0308 (2)	0.000	0.02466 (19)	0.000
F1	0.117 (2)	0.1067 (19)	0.136 (2)	0.0427 (17)	0.101 (2)	0.0050 (17)
O1	0.0378 (9)	0.0428 (9)	0.0360 (9)	−0.0102 (7)	0.0302 (8)	−0.0072 (7)
N1	0.0344 (11)	0.0514 (12)	0.0280 (11)	−0.0005 (9)	0.0230 (10)	−0.0038 (9)
C1	0.0265 (11)	0.0328 (11)	0.0279 (11)	0.0017 (9)	0.0198 (10)	0.0031 (9)
C2	0.0289 (12)	0.0357 (12)	0.0282 (12)	−0.0034 (9)	0.0196 (11)	−0.0021 (9)
C3	0.0388 (13)	0.0420 (12)	0.0345 (13)	−0.0029 (10)	0.0296 (12)	−0.0015 (10)
C4	0.0349 (12)	0.0438 (13)	0.0406 (14)	−0.0041 (11)	0.0303 (12)	0.0030 (11)
C5	0.0342 (13)	0.0404 (12)	0.0356 (13)	−0.0066 (10)	0.0253 (12)	−0.0041 (10)
C6	0.0300 (11)	0.0383 (12)	0.0300 (12)	−0.0030 (9)	0.0222 (11)	−0.0023 (9)
C7	0.0304 (12)	0.0444 (14)	0.0329 (13)	−0.0078 (11)	0.0212 (11)	−0.0106 (11)
C8	0.0353 (14)	0.0686 (17)	0.0274 (13)	0.0040 (13)	0.0207 (12)	−0.0035 (12)
C9	0.0359 (12)	0.0437 (13)	0.0307 (12)	−0.0058 (11)	0.0222 (11)	−0.0080 (11)
C10	0.0658 (19)	0.0631 (18)	0.0575 (18)	0.0062 (17)	0.0469 (17)	0.0110 (16)
C11	0.074 (2)	0.060 (2)	0.083 (3)	0.0224 (18)	0.054 (2)	0.019 (2)
C12	0.061 (2)	0.063 (2)	0.068 (2)	0.0075 (17)	0.0479 (18)	−0.0103 (18)
C13	0.0561 (18)	0.073 (2)	0.0455 (16)	−0.0029 (16)	0.0389 (15)	−0.0098 (16)
C14	0.0401 (14)	0.0511 (15)	0.0349 (13)	0.0004 (12)	0.0260 (12)	−0.0039 (11)

### Geometric parameters (Å, º)

**Table:**

Br1—C2	1.888 (2)	C5—H5	0.9300
Br2—C4	1.902 (2)	C6—C7	1.446 (3)
Zn1—O1	1.9311 (16)	C7—H7	0.9300
Zn1—O1^i^	1.9311 (17)	C8—C9	1.505 (4)
Zn1—N1	2.027 (2)	C8—H8A	0.9700
Zn1—N1^i^	2.027 (2)	C8—H8B	0.9700
F1—C12	1.361 (4)	C9—C10	1.375 (4)
O1—C1	1.299 (3)	C9—C14	1.380 (4)
N1—C7	1.274 (3)	C10—C11	1.390 (5)
N1—C8	1.473 (3)	C10—H10	0.9300
C1—C2	1.412 (3)	C11—C12	1.360 (5)
C1—C6	1.427 (3)	C11—H11	0.9300
C2—C3	1.384 (3)	C12—C13	1.353 (5)
C3—C4	1.392 (4)	C13—C14	1.384 (4)
C3—H3	0.9300	C13—H13	0.9300
C4—C5	1.362 (3)	C14—H14	0.9300
C5—C6	1.408 (3)		
			
O1—Zn1—O1^i^	139.77 (10)	N1—C7—C6	127.3 (2)
O1—Zn1—N1	93.47 (7)	N1—C7—H7	116.3
O1^i^—Zn1—N1	104.97 (8)	C6—C7—H7	116.3
O1—Zn1—N1^i^	104.97 (8)	N1—C8—C9	113.3 (2)
O1^i^—Zn1—N1^i^	93.47 (7)	N1—C8—H8A	108.9
N1—Zn1—N1^i^	124.76 (13)	C9—C8—H8A	108.9
C1—O1—Zn1	123.68 (15)	N1—C8—H8B	108.9
C7—N1—C8	117.5 (2)	C9—C8—H8B	108.9
C7—N1—Zn1	120.79 (17)	H8A—C8—H8B	107.7
C8—N1—Zn1	121.59 (16)	C10—C9—C14	118.9 (3)
O1—C1—C2	119.5 (2)	C10—C9—C8	121.4 (2)
O1—C1—C6	124.7 (2)	C14—C9—C8	119.7 (2)
C2—C1—C6	115.9 (2)	C9—C10—C11	120.7 (3)
C3—C2—C1	123.3 (2)	C9—C10—H10	119.6
C3—C2—Br1	119.44 (18)	C11—C10—H10	119.6
C1—C2—Br1	117.22 (16)	C12—C11—C10	118.3 (3)
C2—C3—C4	118.5 (2)	C12—C11—H11	120.9
C2—C3—H3	120.7	C10—C11—H11	120.9
C4—C3—H3	120.7	C13—C12—C11	122.7 (3)
C5—C4—C3	121.3 (2)	C13—C12—F1	118.6 (3)
C5—C4—Br2	119.04 (19)	C11—C12—F1	118.7 (3)
C3—C4—Br2	119.68 (18)	C12—C13—C14	118.6 (3)
C4—C5—C6	120.4 (2)	C12—C13—H13	120.7
C4—C5—H5	119.8	C14—C13—H13	120.7
C6—C5—H5	119.8	C9—C14—C13	120.8 (3)
C5—C6—C1	120.6 (2)	C9—C14—H14	119.6
C5—C6—C7	116.0 (2)	C13—C14—H14	119.6
C1—C6—C7	123.2 (2)		
			
O1^i^—Zn1—O1—C1	145.92 (19)	O1—C1—C6—C5	179.5 (2)
N1—Zn1—O1—C1	27.73 (19)	C2—C1—C6—C5	−1.4 (3)
N1^i^—Zn1—O1—C1	−99.68 (19)	O1—C1—C6—C7	−5.3 (4)
O1—Zn1—N1—C7	−20.3 (2)	C2—C1—C6—C7	173.8 (2)
O1^i^—Zn1—N1—C7	−164.2 (2)	C8—N1—C7—C6	−172.0 (3)
N1^i^—Zn1—N1—C7	90.6 (2)	Zn1—N1—C7—C6	4.6 (4)
O1—Zn1—N1—C8	156.2 (2)	C5—C6—C7—N1	−171.5 (3)
O1^i^—Zn1—N1—C8	12.3 (2)	C1—C6—C7—N1	13.1 (4)
N1^i^—Zn1—N1—C8	−92.9 (2)	C7—N1—C8—C9	−124.7 (3)
Zn1—O1—C1—C2	161.59 (16)	Zn1—N1—C8—C9	58.7 (3)
Zn1—O1—C1—C6	−19.3 (3)	N1—C8—C9—C10	41.3 (4)
O1—C1—C2—C3	−179.2 (2)	N1—C8—C9—C14	−140.9 (3)
C6—C1—C2—C3	1.7 (3)	C14—C9—C10—C11	0.2 (5)
O1—C1—C2—Br1	−2.1 (3)	C8—C9—C10—C11	178.0 (3)
C6—C1—C2—Br1	178.73 (17)	C9—C10—C11—C12	0.1 (6)
C1—C2—C3—C4	−0.2 (4)	C10—C11—C12—C13	−0.4 (6)
Br1—C2—C3—C4	−177.20 (19)	C10—C11—C12—F1	179.5 (3)
C2—C3—C4—C5	−1.7 (4)	C11—C12—C13—C14	0.5 (5)
C2—C3—C4—Br2	177.87 (19)	F1—C12—C13—C14	−179.4 (3)
C3—C4—C5—C6	1.9 (4)	C10—C9—C14—C13	−0.1 (4)
Br2—C4—C5—C6	−177.63 (19)	C8—C9—C14—C13	−178.0 (3)
C4—C5—C6—C1	−0.3 (4)	C12—C13—C14—C9	−0.2 (4)
C4—C5—C6—C7	−175.8 (2)		

Symmetry code: (i) −*x*+2, *y*, −*z*+2.

## References

[bb1] Cohen, M. D., Schmidt, G. M. J. & Flavian, S. (1964). *J. Chem. Soc.* pp. 2041–2043.

[bb2] Flack, H. D. (1983). *Acta Cryst.* A**39**, 876–881.

[bb3] Higashi, T. (1995). *ABSCOR* Rigaku Corporation, Tokyo, Japan.

[bb4] Rigaku (1998). *RAPID-AUTO* Rigaku Corporation, Tokyo, Japan.

[bb5] Rigaku/MSC (2004). *CrystalStructure* Rigaku/MSC Inc., The Woodlands, Texas, USA.

[bb6] Rodriguez Barbarin, C. O., Bailey, N. A., Fenton, D. E. & He, Q. (1994). *Inorg. Chim. Acta*, **219**, 205–207.

[bb7] Sheldrick, G. M. (2008). *Acta Cryst.* A**64**, 112–122. 10.1107/S0108767307043930 18156677

